# Supplementary material for: Are carbon emissions trading and green financial instruments synergistic? -Comprehensive quantitative research based on content analysis
Source: PLoS One. 2024 Mar 7;19(3):e0298601. doi: 10.1371/journal.pone.0298601 (PMC10919601; doi:10.1371/journal.pone.0298601)
Supplement: S1 Data — (ZIP) [file pone.0298601.s001.zip › Data/地级市Z_F工作报告.zip!/║╙─╧/╚2├┼╧┐╩╨/└┤╫╘╣1⁄2╓┌║┼í╛┬φ┐╦╩2╛▌═°í┐_2013.docx]

各位代表：

　　现在，我代表市人民政府向大会报告工作，请予审议，并请各位政协委员提出意见。

　　一、2012年工作回顾

　　过去的一年，面对经济下行压力加大、困难和挑战增多的复杂形势，市政府在省委、省政府和市委的正确领导下，在市人大、市政协的监督支持下，认真贯彻党的十八大精神，抢抓建设中原经济区、豫晋陕黄河金三角承接产业转移示范区的历史机遇，团结和依靠全市人民，按照“求转、求进、求实”的总要求，围绕“三大战略定位”，扎实推进“四大一高”战略，突出“开转合实”，加快转型发展，完成了市六届人大一次会议确定的目标任务。全市生产总值完成1148.8亿元，比上年增长12.1%；公共财政预算收入完成68.6亿元，增长19.1%；全社会固定资产投资完成937.7亿元，增长23.3%；社会消费品零售总额完成273.2亿元，增长15.8%；出口总值完成1.94亿美元，增长20%。城镇居民人均可支配收入19184元，农民人均纯收入7906元，分别增长12.4%和14.1%。人口自然增长率4.2％……万元GDP能耗下降7%。

　　突出“四大一高”，转型发展成效显着。实施“四大一高”重点项目221个，完成投资575亿元，占年计划的143%，92个项目建成投用。大通关初步形成。三门峡海关即将开关，国家铝及铝制品质检中心通过资质认证，国家果品及果蔬汁检测重点实验室落户我市。大交通建设加快。蒙西铁路建设指挥部即将进驻，三淅高速灵宝至卢氏段、郑卢高速洛宁至卢氏段建成，全市交通投资突破100亿元。大商贸日益繁荣。市商务中心区和湖滨区、陕县、渑池县特色商业区获省批复。金三角建材物流港等大型物流项目建成投用。大中海商业文化广场开业在即，湖滨核心商业圈初步形成。首届中国特色商品博览交易会圆满成功，达成贸易采购合同73亿元，签约重大经济合作协议27个，总金额244亿元，拉动相关产业收入增加18亿元以上，我市被确定为特博会永久举办地。大旅游发展提速。成功举办第十八届旅游节暨投洽会，天鹅湖国家城市湿地公园和函谷关联合创建5A级景区工作全面启动，灵宝汉山、渑池黄河丹峡建成4A级景区。全年接待国内外游客1750万人次，旅游总收入150亿元，分别增长20%和28%。高新技术产业带动能力提升。在全省率先出台《加快发展高新技术产业的十条意见》，实施高新技术重点项目110个，全年完成投资298亿元；高新技术产业完成增加值44.7亿元，增长28.6%。恒生科技公司等4家企业被新认定为高新技术企业，新增院士工作站4家，科技进步对经济发展的贡献率达53.9%。我市“四大一高”引领转型、三化协调科学发展的探索，引起《人民日报》、中央电视台等中央主流媒体的高度关注，走出了属于三门峡的转型之路，谱写了科学发展的华彩乐章。

　　突出“一高两化”，工业经济动力增强。实施亿元以上工业升级项目58个，完成投资132亿元，开祥化工1,4—丁二醇等29个项目建成投产。全市工业投资完成523.6亿元，增长31.1%；规模以上工业增加值完成674.8亿元，增长15%。加快产业集聚区建设，7个省级产业集聚区完成投资388.6亿元，增长43.6%，三门峡产业集聚区建成国家新型工业化示范基地、省级高新技术开发区，三门峡和渑池县产业集聚区建成省高新技术特色产业基地，三门峡和义马市产业集聚区被评为全省“十强”和“十先”产业集聚区。把服务企业作为稳增长的重要措施，建立重点企业监测制度，开辟电煤绿色通道，协调企业加强合作，积极解决电价补贴、要素保障等问题，服务市场主体的水平不断提升。

　　突出引领带动，城乡建设步伐加快。按照“两大三小”总体布局，中心城区建成区面积拓展5平方公里；新建加气站1座，铺设天然气管道13公里；新建换热站4座，新增供热面积20万平方米。三门峡国际文博城和文化公园成为城区新亮点，传媒大厦等城建工程进展顺利。北环路工程主体完工，黄河公园完成建设标段27个，园林形象初步显现。商务中心区建设全面铺开，落地项目30个，完成投资20亿元。新区总体方案正式获批，总体规划开始编制。义渑城区、灵宝城区和卢氏县城建设加快，湖滨区、渑池县、陕县特色商业区完成投资6.8亿元。新开工新型农村社区住宅1.2万套，建成9282套。

　　突出农民增收，特色农业效益提高。培育果品、烟叶、食用菌等农业产业化集群8个，带动农户24万户，实现销售收入122亿元。粮食总产6.6亿公斤，果品总产20.1亿公斤。烟叶收购77.2万担，烟农收入、烟叶税收实现翻番。雏鹰农牧等7个新项目完成投资10亿元，规模养殖场发展到7920个。培育农业龙头企业172家，带动农户33万户，发展农民专业合作社490家。新制定农业生产技术标准27个，市农业物联网总控中心建成。山口水库下闸蓄水，治理水土流失面积81平方公里，新增有效灌溉面积3.4万亩、坡改梯面积3万亩。

　　突出开放合作，发展活力持续显现。豫晋陕黄河金三角承接产业转移示范区上升为国家战略。成功举办了2012中国中小城市发展峰会。全年重大经贸活动签订合作项目177个，签约金额1347亿元；实际利用外资7.5亿美元，增长18.2%；实际到位省外资金220亿元，增长36%。中金集团黄金产业园、大唐三期等12个央企合作项目进展顺利。发挥财税职能，做大融资平台，为经济发展和民生改善提供了资金支持。与省工、农、中、建和农发五大银行签订五年战略合作协议，总金额1075亿元，已到位资金236亿元。广发银行在我市设立分支机构，成立卢氏德丰等村镇银行3家，全市各金融机构新增贷款86.2亿元，增长22.6%，增幅居全省第二。新缔结国际友好城市2对，认真组织“仰韶文化欧洲行”活动，城市知名度和国际影响力不断提升。

　　突出以人为本，人民生活逐步改善。各级财政民生支出98.7亿元，占公共预算支出的72%，省、市“十大民生工程”完成年度任务。城镇新增就业5.1万人，城镇登记失业率2.9%。开展“崤函创业扶持行动”，发放小额担保贷款6.5亿元，其中2.8亿元发放给3800多户小微企业和个体工商户。社会保险覆盖城乡，全市参保人数251万人（次），支付社保资金18.3亿元。城乡低保发放低保金1.96亿元，人均月补助分别比上年提高43元和12元；农村五保集中和分散供养标准分别比上年提高419元、334元，发放供养资金1500万元。新开工保障性住房1.1万套，建成8789套，1822户困难群众拿到了廉租房钥匙。帮助2.7万农村贫困人口脱贫，解决了15万农村人口饮水安全问题。三门峡社会管理职业学院开始筹建，职教园区开工建设，全市新建、改扩建幼儿园49所，15所薄弱学校改造项目开工，改造达标农村学校食堂23个。基层文化设施免费开放，老子文化产业园建成全省文化产业示范基地，三门峡系列电影拍摄完成并试映。全年投入基本卫生服务经费5758万元，人均25元。深入实施国家基本药物制度，减轻群众药费负担1263万元。新农合参合率99.1%，财政补助资金每人增加40元，报销封顶线提高到15万元。实施“健康快车”免费手术1500例。义马铬渣无害化处置完成，淘汰“十五小”企业36家，拆除设施1700余台，实施减排项目107个，城市饮用水源地水质达标率98%以上，成功创建国家森林城市。完善安全生产管理机制，加强食品、药品安全监管，扎实推进社会管理创新，健全信访维稳工作机制，规范信访秩序，社会大局和谐稳定。

　　突出“两转两提”，精神面貌焕然一新。深入开展“两转两提”，从严治理庸懒散软，电子监察系统运行良好。自觉接受人大、政协监督，办理人大代表建议88件、政协提案238件。市文广新局和邮政管理局挂牌，市公共资源交易中心成立。建成市级救灾储备库，应急保障能力不断增强。完成第四届社区居委会换届选举，2个县（市、区）、8个乡（镇）被命名为省级村务公开民主管理示范单位。精神文明建设取得丰硕成果，省级以上文明单位达到155家。在三门峡大地上涌现出了“最美乡村医生”马云飞等一批先进典型，他们身上所展现的“明理诚信、善做善成”的“三门峡精神”，是引领全市发展的精神旗帜和动力源泉！

　　国防教育和国防后备力量建设取得新成效，创造条件支持部队建设，积极推进军民融合式发展，实现了国家双拥模范城“四连冠”，驻三门峡解放军、武警部队和广大预备役官兵在支持地方经济建设中发挥了重要作用。审计、民族、宗教、对台、史志、人防、无线电、黄河河务、妇女儿童、残疾人、老龄等工作取得了新成绩。对口援疆工作进展顺利。

　　各位代表！过去一年，我们在重重困难和挑战面前，交出了一份写满艰辛、载满收获的答卷。这归功于省委、省政府和市委的正确领导，归功于市人大、市政协的监督支持，归功于全市人民的奋力拼搏，归功于社会各界的积极奉献。在此，我代表市人民政府，向辛勤劳动的全市人民，向监督支持政府工作的人大代表、政协委员，向驻三门峡人民解放军、武警官兵、公安民警，向广大客商以及关心支持三门峡发展的各界人士，致以崇高的敬意和诚挚的感谢！

　　同时，我们要清醒认识到，政府工作仍面临一些矛盾和问题：全市转型发展任务艰巨，资金、土地、环境等要素制约依然存在，部分行业的发展困境需尽快摆脱，城乡基础设施还存在不少“欠账”，改善民生和加强社会管理的任务还很繁重，政府行政效率有待进一步提高等。对此，我们必须高度重视，努力加以解决。

　　二、2013年总体要求和主要预期目标

　　今年是全面贯彻落实党的十八大精神、实施《中原经济区规划》、建设豫晋陕黄河金三角承接产业转移示范区的开局之年，是“十二五”规划承前启后的关键之年。按照市委六届四次全会暨市委经济工作会议的部署，今年政府工作的总体要求是：全面贯彻落实党的十八大精神，以邓小平理论、“三个代表”重要思想、科学发展观为指导，坚持五位一体、四化同步，按照省委、省政府的要求，在加快信息化进程中持续探索“两不三新”三化协调科学发展路子，立足我市实际，继续把握好“求转、求进、求实”的总要求，紧紧围绕“三大战略定位”，深入推进“四大一高”战略，以提高经济增长质量和效益为立足点，以“一高两化”为方向，以承接产业转移、新型城镇化引领、优化环境为重点，着力培育形成发展的新动力、新活力、新体系、新优势，稳增长、促转型、惠民生，努力实现经济社会持续健康较快发展。

　　经济社会发展的主要预期目标是：全市生产总值增长11%；公共财政预算收入增长13%；规模以上工业增加值增长15.5%；全社会固定资产投资增长22%；社会消费品零售总额增长16%；实际利用外资增长12%，外贸进出口总额增长15%；城镇居民人均可支配收入和农民人均纯收入均增长10%以上；城镇登记失业率控制在4.5%以内；人口自然增长率控制在6.5‰以内；万元GDP能耗下降3%左右。

　　从内外部形势看，转型道路上仍充满艰辛，我们要保持清醒和忧患，积极寻求转型发展新动力。发展的动力，来自“四大一高”战略的坚强引领和持续推进。历经多年的实践总结，“三大战略定位”坚定不移，“四大一高”引领科学发展，质量和效益成为经济发展新的立足点，我市的发展定位越来越清晰，发展战略越来越科学，发展的路径必须坚持。发展的动力，来自历史机遇的洞察研判和科学把握。党的十八大描绘了全面建成小康社会的宏伟蓝图，中原经济区和黄河金三角示范区搭建了区域发展的广阔平台，全市面临纳入全球分工体系、扩大出口、加快投资的传统机遇，也面临扩大内需、提高创新能力、促进经济发展方式转变的新机遇，发展的历史机遇不容错过。发展的动力，来自省委、省政府的殷切关怀和正确领导。省委、省政府结合中原经济区建设实际，强调突出“四着力”，抓好“三重点”，继续推进“稳增长、促转型、保态势行动计划”，为我市加快转型发展指明了方向。发展的动力，来自多年积累的基础优势和综合实力。通过历届班子的不懈努力，全市主导产业日益壮大，正在向产业链终端和价值链高端延伸。大交通、大通关等基础支撑逐步增强，产业集聚区、商务中心区、特色商业区等发展载体不断完善，经济增长的动力加速形成。发展的动力，来自全市人民的团结奋斗和埋头苦干。在去年的工作推进中，全市上下凝心聚力、苦干实干，谋发展、促转型的氛围日趋浓厚，加快发展的正能量不断凝聚。各位代表，只要我们用好新机遇，再添新动力，就一定能够开创三门峡转型发展的光明前景！

　　三、2013年的重点工作

　　（一）强化“四大一高”战略，加快经济转型发展。强化开放带动，持续完善大通关。健全海关监管堆场等配套设施，积极申报综合保税区，加快国家果品及果蔬汁检测重点实验室和河南省黄金质检中心建设。依托出口基地建设，巩固果汁等优势产品出口，培育食用菌、专用汽车等新的出口增长点，实现出口产品多元化。

　　完善基础支撑，持续推进大交通。加快推进连霍高速改扩建、三淅高速南段等项目，三灵快速通道建成通车。抓好蒙西铁路、芮灵黄河大桥河南段及连接线、渑垣高速等项目前期工作，确保全市交通投资完成80亿元以上。

　　扩大消费需求，持续繁荣大商贸。以办好第二届特博会为抓手，发展会展经济，壮大现代服务业，形成内需拉动效应。开工建设义马煤炭储备基地等一批重大商贸、物流项目。加快现代金融业发展，支持三门峡银行“走出去”，加快农村信用社改制，积极引进域外金融机构入驻。

　　整合优势资源，持续提升大旅游。大力促进文化与旅游要素融合，整合文化山水资源，组建市旅游集团，促进旅游集群发展。深化沿黄九省（区）旅游联盟合作，办好第八届中博会分会场暨第十九届黄河旅游节。推进天鹅湖国家城市湿地公园和函谷关联合创建5A级景区工作，启动仰韶文化博物馆、双龙湾等创建4A级景区，提升旅游核心竞争力。

　　实施创新驱动，持续发展高新技术产业。拓展创新平台，新建院士工作站2家以上，认定省级工程技术研究中心和企业技术中心10家以上。围绕核心关键技术，新实施省级重大科技专项5项，突破重大关键技术10项。抓好速达纯电动汽车、恒生科技丙尔金、义腾新能源二期等项目。

　　充分发挥信息化在推进“四大一高”战略中的带动和提升作用，逐步实现与“三化”融合发展。引导工业企业积极实施信息化项目，以信息化提升企业管理水平和创新能力。抢抓河南省创建全国农村信息化示范省的机遇，持续加快农村信息化建设。加快数字城市、智慧城市建设，大力推进商贸、物流、旅游信息化，让信息化为转型发展注入强大动力。

　　（二）强化工业主导地位，扎实推进“一高两化”。抓好重大工业项目，加快转型升级。确定亿元以上工业项目52个，总投资466亿元，年计划完成投资123亿元。争取戴卡200万只旋压轮毂等23个新建项目上半年开工，确保黄河铝业20万吨铝板一期等29个续建项目年内投产。实施“一高两化”，打造五个千亿元产业集群。依托优势产业，用5年左右时间，打造黄金、铝工业、煤化工、以清洁镀金为主的新材料工业、汽车及零部件等装备制造业五个千亿元产业集群，成为支撑转型发展的核心增长极。今年重点抓好中金集团黄金产业园、同人铝业10万吨高档工业铝合金铸件、骏通公司5万辆专用车、义煤集团1000万标方煤制气及20万吨甲醇蛋白等龙头项目，加快推进新华水工机械公司迁建项目前期工作。统筹抓好食品、医药等新兴产业发展。提升综合带动能力，打造五个百亿元产业集聚区。突出“四集一转”，加快基础设施建设，全面提升综合配套能力。围绕主导产业“招大引强”，每个产业集聚区年内引进固定资产投资超过5亿元的重点工业项目2个以上。年末，全市主营业务收入超百亿元的产业集聚区达到5个，其中义马市产业集聚区超过500亿元，三门峡、渑池县产业集聚区力争达到300亿元，灵宝市产业集聚区超过200亿元，三门峡经济技术产业集聚区超过100亿元，陕县、卢氏县产业集聚区分别超过30亿元和20亿元，湖滨区专业园区达到25亿元。扶持企业做大做强，打造一批大型企业集团。围绕铝工业、黄金及有色金属、煤化工等传统产业，以及新能源汽车、新材料等战略新兴产业，谋划一批大项目，培育大型企业集团。抢抓国家九大行业兼并重组的机遇，支持我市企业与全省、全国骨干企业对接，促进企业做大做强。强化工业项目和经济运行服务。加强对建成项目的协调服务，确保国华公司20万套轨道交通设备等100个竣工项目尽快达产达效。研究出台扶持优质企业的有关政策，全力支持企业发展。继续抓好工业经济运行监测分析，强化要素保障，保持工业经济平稳运行。

　　（三）强化新型城镇化引领，促进城乡一体化发展。把新型城镇化作为扩大投资、拉动消费、改善民生、引领转型的全局性、战略性举措，加快城乡一体化。有序推进农业转移人口市民化。坚持“一基本两牵动”，有序推进中心城区规划区和产业集聚区农村人口市民化。增强产业支撑能力，推动以产兴城，解决好进城农民就业问题。落实国家房地产调控政策，增加普通商品房供应，逐步把具备条件的农民纳入城镇住房保障体系。加强城镇学校建设，解决好进城务工人员的居住及子女入学问题。完善城镇布局和规划。按照“两大三小”组团式布局，完成三门峡市城市总体规划修编，启动中心城区综合交通、供排水等专项规划和区域功能设计。完善县域村镇体系规划、社区布局规划，完成全市139个新型农村社区的空间发展规划和详细规划。提升中心城区功能。加快推进沿黄半岛景观带、北环路等重点城建项目，黄河公园在旅游节前开园。完成市污水处理厂搬迁，完善城区雨污水管网、垃圾中转站等基础设施，启动华阳电厂换热首站和主管网建设。扎实推进新区工作。加快编制新区总体规划，积极研究制定招商、融资等配套政策。全面掀起商务中心区建设高潮。完成基础设施投资20亿元以上，项目投资80亿元以上，新落地商务、商业项目5个以上，中心大道、甘棠南路等8条道路建成，明珠广场基本完工，实现商务中心区“两年出形象”的目标。加快特色商业区建设。以城中村和旧城改造为载体，加快湖滨区特色商业区建设，华创国际广场一期封顶，嘉亿城市广场完成投资8亿元以上。义马市、灵宝市和卢氏县特色商业区规划争取上半年获批，加快各县（市）特色商业区项目建设，再落地一批新项目，使商务中心区和特色商业区成为带动城区发展的新动力。加快新型农村社区建设。新开工新型农村社区住宅1万套以上，初步建成10个规模集中型重点社区。

　　（四）强化农业基础支撑，增强农村经济活力。全面落实各项惠农政策，扎实推进新型农业现代化。壮大农业产业化集群。推进高标准生产基地建设，继续壮大果品、烟叶等8个农业产业化集群，培育市级以上农业龙头企业23个。新栽苹果20万亩，力争果品总产稳定在20亿公斤。种植烟叶30万亩，收购量继续保持全省第一。发展食用菌1.4亿袋、蔬菜9亿公斤、水产品1.9万吨。健全现代农业支撑和服务体系。继续推进农业标准化建设，建成全国农业综合标准化示范市。加强农业科技、质量安全、市场流通等公共服务体系建设，力争年内实现县级农产品质检中心全覆盖。加强防汛预警体系、农村气象灾害防御体系建设，提高防灾减灾能力。培育新型农业经营主体。有序推进土地流转，扶持联户经营、专业大户、家庭农场，加快发展新型农民合作组织。依托农业龙头企业，培训新型农民和农村实用人才，完成“农村劳动力转移技能就业计划”培训2万人，阳光工程培训9000人，全年劳务输出35万人次。改善农村生产生活条件。抓好白虎潭水库、山口河治理等项目，做好大石涧水库、鸡湾水库前期工作，新建改建县乡道路200公里，加快新一轮农网改造升级，再解决10万农村群众饮水安全问题。落实国家扶贫开发政策，再帮助2万农村贫困人口稳定脱贫。

　　（五）强化投资拉动，着力抓好重点项目建设。今年安排重点项目300个左右，总投资2000亿元以上，年计划完成投资500亿元以上。加快项目进度。强化项目服务和管理，继续实行重大项目领导分包责任制、联审联批、周例会等制度，全力加快项目建设。对192个在建项目，抓好跟踪调度和服务保障，确保尽快竣工投产。对115个具备开工条件的项目，加快手续报批，完善配套服务，力争 10月前全部开工。优化项目结构。集中精力推进大项目，重点抓好拉动就业、促进产业结构调整的项目。坚持以项目促发展，抓好100个重大工业升级和结构调整项目，抓好一批新型城镇化、现代服务业、交通、农林水、民生、环保等项目。做好新项目谋划。围绕国家产业政策，完善重点项目遴选机制，年内新谋划工业前期项目150个以上，确保50个项目当年完成前期工作。

　　（六）强化改革创新，激发科学发展动力。加大改革创新，释放发展的巨大潜力、源头活力和持久动力。深入推进重点领域改革。深化农村改革，推进集体林权、国有林场和供销社改革，争取进入省农村金融改革试点。积极稳妥推进公立医院改革和基层医疗卫生机构综合改革。加快推进财政、金融、价格、事业单位、文化体制等其他领域的改革。创新要素保障机制。实施“崤函人才计划”，为转型发展提供强大的智力支持。围绕中央、省资金投向，积极谋划项目、争取投资。落实好与五大银行的战略合作协议。增强各级投融资平台实力，设立直接债务融资发展基金，加快推进“区域集优”直接债务融资。强化节约集约用地，扎实开展土地综合整治，严格落实城镇和产业集聚区建设用地标准。引导企业依法转让或出租闲置厂房，探索建立企业用地退出机制。加快非公有制经济发展。支持民间资本参与重大基础设施和公益性项目建设，实施中小微企业成长工程，落实小额担保贷款等支持企业发展政策，解决好中小微企业贷款难问题，促进非公有制经济加快发展。鼓励支持企业上市。积极开展企业上市培训，帮助企业加强与创投和私募基金的对接，引进战略投资者。出台扶持政策，完善激励机制，加快金源矿业等9家省上市后备企业上市步伐。

　　（七）强化开放招商，优化经济发展环境。突出开放招商“一举求多效”，以开放促发展、促转型。扎实开展大招商。精心办好第八届中博会分会场暨旅游节等招商活动，确保全市招商签约金额继续保持在1000亿元以上，利用省外资金增长20%。注重招商引资实效。发挥豫晋陕黄河金三角承接产业转移示范区的优势，研究国内外产业转移趋势、央企和大企业战略布局，紧盯产业转移重点区域，围绕现有企业的原材料供应、零部件配套、产品销售等链条，开展专业化集群招商。狠抓招商项目落地。加强签约项目的跟踪督导，实行“保姆式”服务，切实提高项目的履约率、开工率和资金到位率。优化经济发展环境。坚持优化环境“一优带百通”，精简行政审批事项，加强行政服务中心建设，优化招商服务环境。加强诚信体系建设，优化金融生态环境。严厉打击扰乱项目施工等违法行为，优化项目施工环境。严禁任何单位和个人擅自对企业进行检查、收费、罚款、评比等行为，优化企业生产经营环境。

　　（八）强化生态文明建设，打造美丽三门峡。加强生态环境治理。深入实施“碧水蓝天美丽家园345工程”，通过3个水污染治理工程、4个大气污染治理工程、5个生态创建工程，提升生态环境质量。开展机动车污染防治，依法取缔市区、陕县城区燃煤小锅炉，启动PM2.5监测评价体系。完成4个污水处理厂升级改造和8个污水处理厂新建任务。抓好节能减排。实行能源消费总量和污染物排放总量预算管理，探索实施排污权有偿使用和交易。继续推动实施节能减排重点工程，大力发展节能减排先进技术，降低资源能源消耗强度。严格环保准入和污染物排放标准，强化工程减排、结构减排和管理减排。加强农村环境连片综合整治，控制面源污染。大力发展绿色、低碳、循环经济。抓好金源矿业30万吨黄金冶炼废渣综合利用等循环经济项目，加快渑池县国家循环经济标准化试点县建设，努力创建一批国家级循环经济示范县（市）、园区和企业。巩固生态建设成果。扎实推进天然林保护、退耕还林、农户造林、森林抚育试点等项目，完成生态造林31.8万亩，特色经济林15万亩。积极创建全国绿化模范市，建成省级生态乡镇5个、省级生态村20个。无论任何时候，我们都将始终坚持尊重自然、顺应自然、保护自然，把生态文明建设融入到经济社会发展的全过程，让三门峡青山常在、绿水长流。

　　（九）强化民生改善，持续创新社会管理。继续实施“十大民生工程”，让群众得到更多实惠。推动实现更高质量的就业。把就业作为最大的民生，重点做好大中专毕业生、农村转移劳动力、城镇困难人员、退役军人就业工作。持续推进“崤函创业扶持行动”，全年发放小额担保贷款5亿元以上，以创业带动就业。全年新增就业4万人以上，失业人员再就业2万人。积极推进城乡社会保障体系建设。推动城镇职工养老保险与城乡居民养老保险衔接，城镇职工医保与居民医保、新农合衔接，继续提高城镇居民医保、新农合财政补助标准和实际报销水平。规范完善基本养老保险省级统筹，实施失业保险市级统筹。继续提高企业退休人员基本养老金水平，提高城乡低保、农村五保供养标准和优抚对象补助水平。完善社会救助、社会福利制度，大力发展老龄、残疾人、慈善事业。新开工各类保障性住房8600套，确保工程质量、公平分配、动态管理、有效入住。提高教育发展水平。做好社会管理职业学院筹建工作，年内通过评估验收，加快职教园区项目和校舍安全工程建设，全面完成学前教育三年行动计划。加大文化惠民力度。以老子、仰韶文化产业园和仰韶文化研究中心为抓手，加快文化产业示范基地建设。扶持文化龙头企业，打造知名文化品牌，促进文化产业发展。抓好主题文化公园、传媒大厦等文化惠民工程，加大对农村文化阵地建设扶持力度，构建覆盖城乡、惠及全民的公共文化服务体系。提高人民健康水平。做好市儿童医院、市妇幼保健院等项目前期工作，争取市中心医院、黄河医院通过三甲医院验收，市中医院通过三甲中医院验收。积极实施国家基本公共卫生服务项目，建设覆盖城乡的基本医疗卫生体系。完善人口和计划生育政策服务体系，稳定低生育水平。广泛开展群众性体育运动，办好市第七届运动会。持续加强和创新社会管理。改进政府提供公共服务方式，完善村（居）民自治制度，健全社会管理服务网络。做好群众工作，畅通群众诉求表达、利益协调、权益保障渠道。加强应急管理，提高突发事件处置应对能力。完善食品、药品安全监管体制，强化安全生产监管，确保安全形势平稳。深入推进平安三门峡建设，争创全国综合治理优秀市“三连冠”，努力夺取“长安杯”。

　　继续加强国防动员、国防教育和国防后备力量建设，支持驻三门峡解放军、武警部队、民兵、预备役和人防建设，深入开展双拥共建，巩固军政军民团结。继续做好统计、外侨、对台、民族、宗教、史志、防震减灾等工作。扎实做好对口援疆工作。

　　各位代表，全市人民对美好生活的向往，就是我们政府工作的努力方向。我们要从群众最关心的事情抓起，从群众最盼望的事情做起，量力而行，尽力而为，努力让城乡居民的生活过得一年更比一年好！

　　四、加强政府自身建设

　　全面贯彻中央“八项规定”，落实“三具两基一抓手”工作方法和“四个明白”工作要求，深入推进“两转两提”，牢固树立为民负责、推动工作、清正廉洁的政府形象。

　　牢记为民宗旨。始终把人民放在心中最高位置，真诚倾听群众呼声，真情关心群众疾苦，真心为民排忧解难。强化社会管理和公共服务职能，把更多的人力、物力、财力投向民生领域，让改革发展的成果惠及全市人民。

　　提高行政效能。准确判断、正确应对形势变化，充分发扬民主，健全决策规程，不断提高科学决策能力。善于统筹兼顾，抓大事、办急事、解难事，不断提高运作能力。善于把上级精神与本地实际相结合，克服困难、解决问题，狠抓落实，不断提高政府执行力，促进各项工作提速提效。

　　坚持依法行政。继续执行好政府领导带头学法制度，依法依规调节经济运行和社会事务。深入开展法制宣传教育，治理行政执法中存在的突出问题，增强公务人员依法行政意识。严格执行市人大及其常委会的决议决定，认真办理人大代表建议和政协提案。自觉接受人大的法律监督、工作监督，接受市政协和各民主党派的民主监督，接受社会和舆论监督，让政府更好地服务人民。

　　改进工作作风。牢记实干兴邦，说实话、办实事、勇担当，把精力用到谋发展、抓落实、干事业上。针对薄弱环节，实施重点突破，带着问题到基层，带着办法见群众，研究措施、解决困难、推进工作。认真落实党风廉政建设责任制，加强监察、审计监督，完善惩防体系，规范权力运行；厉行勤俭节约，狠刹浪费之风，做到干部清正、政府清廉、政治清明。

　　各位代表！我市的转型发展已经进入了关键阶段，蓝图引领方向，实干成就梦想。让我们在省委、省政府和市委的坚强领导下，砥砺奋进，克难攻坚，为加快建设创新开放、富裕文明、平安和谐、生态宜居三门峡而努力奋斗！
